# Masitinib as a neuroprotective agent: a scoping review of preclinical and clinical evidence

**DOI:** 10.1007/s10072-023-07259-w

**Published:** 2023-12-18

**Authors:** Abdullah Ashraf Hamad, Basma Ehab Amer, Yousef Hawas, Manar Alaa Mabrouk, Mostafa Meshref

**Affiliations:** 1 Faculty of Medicine, Menoufia University, Menoufia, Egypt; 2 Faculty of Medicine, Benha University, Benha, Egypt; 3 Faculty of Medicine, Tanta University, Tanta, Egypt; 4 Faculty of Medicine, Fayoum University, Fayoum, Egypt; 5 Medical Research Group of Egypt, Negida Academy, Arlington, MA USA; 6 Department of Neurology, Faculty of Medicine, Al-Azhar University, Cairo, Egypt

**Keywords:** Masitinib, Neurodegeneration, Neuroprotection, ALS, Alzheimer’s disease, Multiple sclerosis

## Abstract

**Objectives:**

Masitinib, originally developed as a tyrosine kinase inhibitor for cancer treatment, has shown potential neuroprotective effects in various neurological disorders by modulating key pathways implicated in neurodegeneration. This scoping review aimed to summarize the current evidence of masitinib’s neuroprotective activities from preclinical to clinical studies.

**Methods:**

This scoping review was conducted following the guidelines described by Arksey and O’Malley and the Preferred Reporting Items for Systematic Reviews and Meta-Analyses guidelines. The inclusion criteria covered all original studies reporting on the neuroprotective effects of masitinib, including clinical studies, animal studies, and in vitro studies.

**Results:**

A total of 16 studies met the inclusion criteria and were included in the review. These comprised five randomized controlled trials (RCTs), one post-hoc analysis study, one case report, and nine animal studies. The RCTs focused on Alzheimer’s disease (two studies), multiple sclerosis (two studies), and amyotrophic lateral sclerosis (one study). Across all included studies, masitinib consistently demonstrated neuroprotective properties. However, the majority of RCTs reported concerns regarding the safety profile of masitinib. Preclinical studies revealed the neuroprotective mechanisms of masitinib, which include inhibition of certain kinases interfering with cell proliferation and survival, reduction of neuroinflammation, and exhibition of antioxidant activity.

**Conclusion:**

The current evidence suggests a promising therapeutic benefit of masitinib in neurodegenerative diseases. However, further research is necessary to validate and expand upon these findings, particularly regarding the precise mechanisms through which masitinib exerts its therapeutic effects. Future studies should also focus on addressing the safety concerns associated with masitinib use.

## Introduction

Neurological and neurodegenerative diseases include a broad spectrum of disorders, which affect the central and peripheral nervous systems. They are often associated with functional impairments and diminished quality of life [1]. The prevalence of multiple neurological diseases is increasing [2, 3]. For instance, it is expected that the worldwide prevalence of amyotrophic lateral sclerosis (ALS) will reach 376,674 patients in 2040 with an estimated 69% growth from 222,801 patients in 2015 [2]. Hence, appropriate management strategies are essential to reduce the morbidity and mortality associated with these debilitating disorders. The management of these disorders depends on supportive care, rehabilitation, lifestyle modifications, and pharmacological interventions, which aim to alleviate symptoms, slow disease progression, and consequently minimize disability and improve overall functioning and well-being [4]. Despite the recognized importance of appropriate management strategies, the management of neurological and neurodegenerative diseases encounters various challenges, such as high healthcare costs and limited treatment options for some complex diseases [5].

The exact pathophysiology of neurodegenerative disorders is not fully understood; however, previous research suggested that chronic inflammation largely contributed to the development and progression of these diseases [6]. Given that inflammation of the central nervous system is tightly regulated by astrocytes and microglia, it is hypothesized that manipulation of these cells may provide a possible therapeutic option against these diseases [7]. Therefore, researchers have explored the potential therapeutic benefits of masitinib, an oral selective tyrosine kinase inhibitor that was originally developed as an anticancer drug, in various neurodegenerative disorders, such as Alzheimer’s disease (AD), ALS, and multiple sclerosis (MS) [8–10]. Indeed, preclinical and clinical studies have shown promising results regarding the efficacy of masitinib in neurodegenerative disorders via inhibition of microglia, astrocytes, and mast cell activity in both central and peripheral nervous systems [9–11]. However, randomized controlled trials (RCTs) investigating the efficacy of masitinib in these disorders are still limited, and further well-designed RCTs are warranted to validate the current evidence. In addition, it was also hypothesized that masitinib may improve the prognosis of ischemic stroke because mast cells may participate in the development of ischemic stroke. Therefore, animal studies have been conducted to test this hypothesis [12].

Interestingly, these studies have shown promising results, suggesting that masitinib may be an effective adjunct agent in stroke management. Importantly, the potential benefits, safety profile, and different mechanisms of action of masitinib in all aforementioned neurological diseases have not been summarized in previous reviews. Therefore, we conducted this scoping review to summarize the available evidence regarding the role of masitinib as a neuroprotective agent.

## Methods

### Protocol and registration

This scoping review was conducted following the guidelines described by Arksey and O’Malley and the Preferred Reporting Items for Systematic Reviews and Meta-Analyses (PRISMA) guidelines [13, 14]. Also, the final results are reported according to PRISMA and PRISMA-Scoping Review guidelines [13, 15]. This review was registered in the PROSPERO international prospective register of systematic reviews (registration number CRD42023457214).

### Search strategy

PubMed, Scopus, Web of Science, and Cochrane CENTRAL databases were searched up to August 1, 2023. To ensure a comprehensive search, we focused on using masitinib-related terms exclusively and did not include any neurological terms (“Masitinib” OR “AB1010” OR “Kinavet” OR “Masivet”). No filters or language restrictions were applied. The references of the included studies were also screened to ensure all relevant articles were covered.

### Inclusion criteria

The objective of this scoping review was to summarize the current evidence of the neuroprotective activities of masitinib from preclinical to clinical studies. Thus, our review included all original studies reporting on the neuroprotective effects of masitinib. There were no restrictions placed on the studied disease, population, or outcomes. The inclusion criteria covered (a) RCTs, observational studies, case reports, and case series; (b) animal studies; and (c) in vitro studies. Reviews, editorials, and studies on non-neurological diseases were excluded.

### Study selection and data extraction

Without removing duplicates, two reviewers independently screened the titles and abstracts of the citations [16]. Then, a third reviewer retrieved and screened the full text of the identified studies to make the final decision. After identifying the included studies, two authors extracted the data using an online data extraction form. For RCTs, the extracted data included information such as country, population, sample size, outcomes, and main findings. For animal studies, the extracted data encompassed the animal model, sample size, age, sex, outcomes, and main findings. Any discrepancies in the screening process or data extraction were resolved through discussion with a third author.

### Quality assessment

We followed the Cochrane risk-of-bias tool for randomized trials (RoB 2.0) tool to assess the quality of the included RCTs [17]. This tool evaluates the risk of bias in five domains, including bias arising from the randomization process, deviations from intended interventions, missing outcome data, measurement of outcomes, and selection of the reported result. The trial was considered to be at high risk if at least one domain was rated as high risk, and low risk if all domains were judged as low risk. For animal studies, the CAMARADES checklist was used to assess their quality [18]. There was one case report included and was assessed using the Joanna Briggs Institute tool [19].

### Analysis

Given the heterogeneity in the included studies in terms of populations and outcomes, we conducted a qualitative analysis of the data following the recommended methodology for qualitative reviews outlined in the Cochrane Handbook [20]. We categorized the manuscript into two groups: (i) preclinical and (ii) clinical. For the preclinical studies (animal studies), we tabled the following information: (1) animal model utilized (e.g., mouse, pig); (2) sample size; (3) age, sex, and weight of the animals; (4) outcome measures assessed; (5) main findings; and (6) study quality evaluation. Regarding the clinical studies, we gathered the following details: (1) study design (e.g., RCT, case study); (2) characteristics of the study population (i.e., age and disease condition); (3) outcome measures examined; (4) effects of masitinib on the outcome measures; and (5) evaluation of study quality using the aforementioned criteria.

## Results

### Characteristics of the included studies

**Fig. 1:**
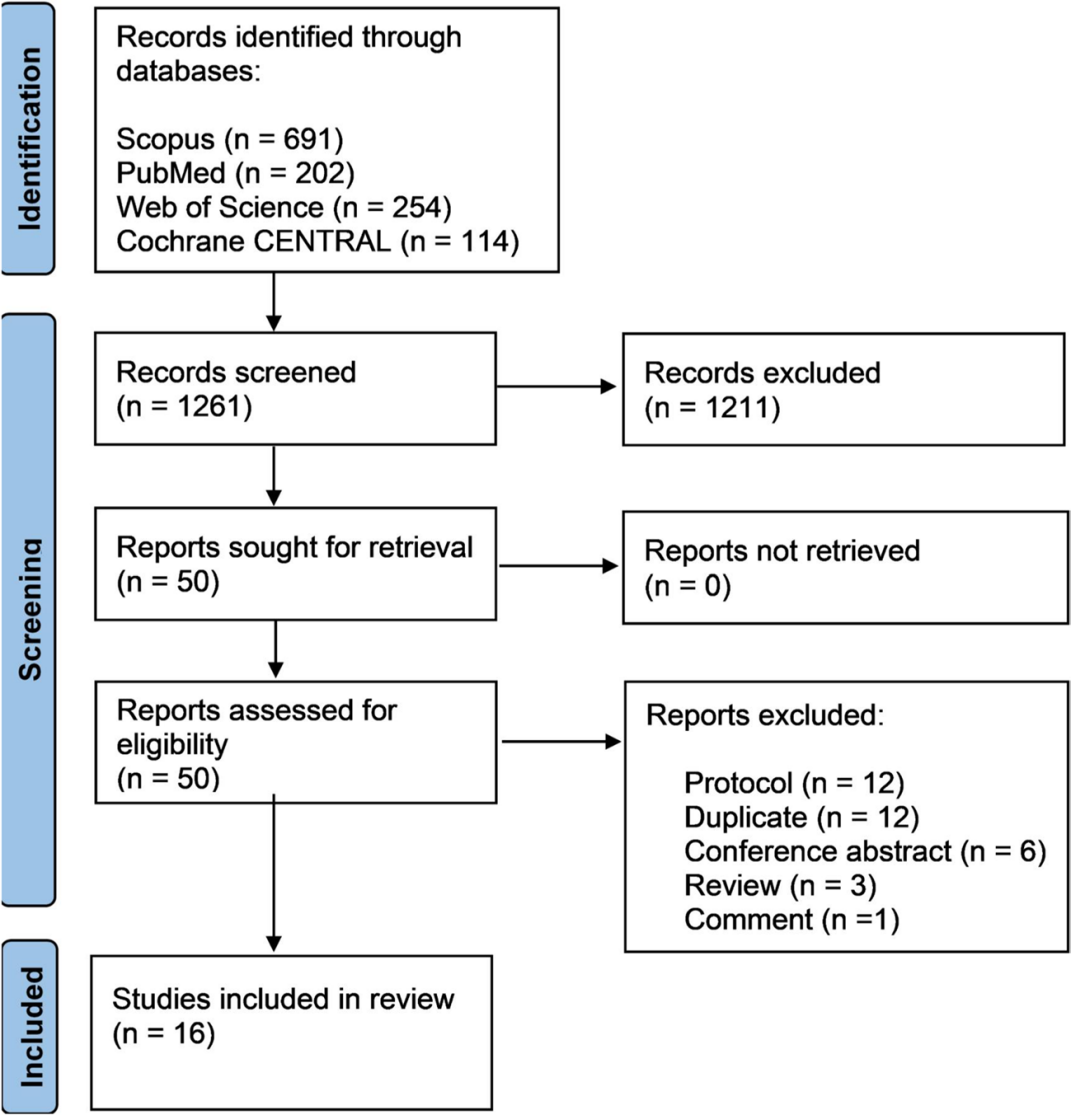
The PRISMA flow diagram

Our search resulted in a total of 1261 citations. After screening the titles and abstracts, 50 records were identified and assessed for eligibility. Of which, 12 were protocols, 12 were duplicates, six were conference abstracts, three were reviews, and one article was an erratum on an included article. Finally, a total of 16 studies met the inclusion criteria and were included in our review. These consisted of five RCTs [8–10, 21, 22], one post-hoc analysis study [23], one case report of a patient included in an RCT [24], and nine animal studies [8, 11, 12, 25–30]. Figure 1 summarizes the selection process of the included studies. The quality of the included RCTs was generally high, as summarized in Table 1. Table 2 summarizes the quality assessment of the included animal studies, which were of moderate quality. The included case report was of good quality based on the Joanna Briggs Institute tool [24].

**Table 1:** Risk of bias assessment of RCTs using the RoB 2.0 tool

Study ID	D1	D2	D3	D4	D5	Overall
Vermersch 2022 [22]	Low	Low	Low	Low	Low	Low
Vermersch 2012 [8]	Some concerns	Low	Low	Low	Some concerns	Some concerns
Piette 2011 [21]	Low	Low	Low	Low	Low	Low
Dubois 2023 [10]	Some concerns	Low	Low	Low	Low	Some concerns
Mora 2019 [9]	Some concerns	Low	Low	Low	Low	Some concerns

*D1*, bias arising from the randomization process; *D2,* bias due to deviations from intended intervention; *D3*, bias due to missing outcome data; *D4*, bias in measurement of the outcome; *D5*, bias in selection of the reported result

**Table 2:** Quality assessment of the animal studies using CAMARADES checklist

Study ID	1	2	3	4	5	6	7	8	9	10	11	12	13	14	Score
Kocic 2015 [12]	Yes	Yes	NR	NR	NR	NR	Yes	NR	Yes	Yes	Yes	NR	NR	Yes	7
Li 2020 [11]	Yes	NR	NR	NR	NR	NR	Yes	NR	Yes	Yes	Yes	NR	NR	Yes	6
Qian 2021 [25]	Yes	Yes	Yes	NR	NR	NR	Yes	NR	Yes	Yes	Yes	NR	Yes	Yes	9
Trias 2016 [26]	Yes	NR	Yes	NR	NR	NR	Yes	NR	Yes	Yes	Yes	Yes	NR	Yes	8
Vermersch 2012 [8]	Yes	NR	NR	NR	NR	NR	Yes	NR	Yes	Yes	Yes	NR	NR	Yes	6
Harrison 2020 [30]	Yes	Yes	NR	NR	NR	NR	Yes	NR	Yes	Yes	Yes	NR	NR	Yes	7
Trais 2017 [27]	Yes	Yes	Yes	NR	NR	NR	Yes	NR	Yes	Yes	Yes	NR	NR	Yes	8
Trais 2018 [28]	Yes	Yes	Yes	NR	NR	NR	Yes	NR	Yes	Yes	Yes	NR	NR	NR	7
Trais 2020 [29]	Yes	Yes	Yes	NR	NR	NR	Yes	NR	Yes	Yes	Yes	NR	NR	NR	7

Studies fulfilling the criteria of (1) peer reviewed publication; (2) control of temperature; (3) random allocation to treatment or control; (4) blinded induction of ischemia; (5) blinded assessment of outcome; (6) use of anesthetic without significant intrinsic neuroprotective activity; (7) animal model with neurodegenerative disease; (8) sample size calculation; (9) compliance with regulatory requirements; (10) statement of potential conflict of interests; (11) physiological monitoring; (12) prespecified inclusion and exclusion criteria; (13) reporting animals excluded from analysis; and (14) reporting of study funding. *NR*, not reported

### Evidence from the preclinical studies

Our comprehensive review identified nine preclinical studies with over 350 animals. Five studies were conducted on SOD1G93A mutant rats modeling ALS, while two studies were on C57BL/6 mice (Table 3). In their study on Wistar rats with post-ischemic stroke, Kocic et al. investigated the effect of masitinib (25 or 100 mg/kg twice daily) on reducing the infarct size and the neurological deficit after the stroke [12]. They found that masitinib alone significantly reduced the infarct size compared with the control group, and masitinib combined with tissue-type plasminogen activator was superior to tissue-type plasminogen activator alone. Masitinib also reduced the neurological symptoms compared to the control group. A similar study by Qian et al. investigated the effects of masitinib on the mechanoreception of sensory neurons in C57BL/6 mice of tourniquet-induced hind paw ischemia–reperfusion [25]. Masitinib mitigated nerve damage and improved hind paw mechanoreception to mechanical stimulation. Trias et al. investigated masitinib in a SOD1G93A mutant rats model of ALS to explore its therapeutic effects and its effects on isolated cultured aberrant glial cells [26]. They found that administration of masitinib decreased aberrant glial cells, improved motor neuron pathology, and prolonged post-paralysis survival. Similar findings were found in other studies, showing improved reinnervation and reduced regressive changes of Schwann cells [27, 30]. Another study was on a mice model of AD by Li et al., investigating the effects of masitinib on cognitive function, neuroinflammation, brain amyloidosis, and synaptic integrity [11]. Masitinib reduced the synaptic integrity and the cognitive anomalies; however, they observed no benefits regarding inflammatory mediators and microglial densities. Besides their RCTs on patients with MS, Vermersch et al. also explored the effects of masitinib using a myelin oligodendrocyte glycoprotein murine model, and found a significant reduction in disease, as assessed by the mean clinical score [8].

**Table 3:** Summary of the included animal studies

Study ID	Animal model	Sample size	Age, sex, weight	Outcomes (in terms of masitinib)	Findings
Kocic 2015 [12]	Wistar rats modeling post-ischemic stroke	56	Elderly rats, male, 270–350 g	Efficacy of masitinib (25 or 100 mg/kg twice daily) alone or in combination with rt-PA or rt-PA alone in reducing infarct size and neurological deficit after induced ischemic stroke	Masitinib alone showed a statistically significant infarct size reduction compared with the stroke control group; brain ischemic area decreased from 9.14 to 4.36% (25 mg/kg) or 2.60% (100 mg/kg) Combined therapy was superior to rt-PA alone in reducing infarct size Masitinib given alone reduced neurological symptoms of stroke particularly if administered at a dose of 100 mg/kg
Trias 2016 [26]	SOD1G93A mutant rats modeling ALS	52	27 males and 25 females	Effects of masitinib (30 mg/kg/day) on cultured aberrant glial cells and determine its therapeutic effect in ALS	Neuroinflammation associated with ALS progression was modulated. Microgliosis and aberrant glial cells were reduced Post-paralysis survival rate increased in both genders by 40%
Li 2020 [11]	Transgenic APPPS1dE9 mice modeling AD	37	12 month old, males	Effects of masitinib (75 mg/kg/day) on cognitive function, neuroinflammation, brain amyloidosis, and synaptic integrity	There was improvement of the cognitive performance in the masitinib group, estimated by the Morris water maze task No difference was noticed between the vehicle and masitinib group regarding the accumulation of aggregated amyloid-β and the pro-inflammatory IL-1β cytokine. There was a significant increase of 20% in synaptophysin immunoreactivity in the masitinib-treated group
Qian 2021 [25]	C57BL/6 mice modeling tourniquet-induced extremity ischemia–reperfusion	174	7–8 weeks of age, 22–24 g	Effect of masitinib on mechanoreception and alteration of sensory nerves during tourniquet-induced ischemia–reperfusion	Reduced ROS production led to mechanoreception improvement and alleviation of sensory nerve damage There were alleviated allodynia and decreased inflammatory cytokines in the masitinib-treated group
Vermersch 2012 [8]	C57BL/6 mice modeling myelin oligodendrocyte glycoprotein-induced experimental allergic encephalomyelitis disease	15	-	Effect of masitinib on the inhibition of mast cell function and the reduction of disease	Treatment with masitinib led to a significant reduction in disease, as assessed by the mean clinical score, when compared with vehicle alone (*P* < 0.001)
Harrison 2020 [30]	hemizygous B6.Cg-Tg(SOD1-G93A)1Gur/J (SOD1G93A) mice modeling ALS	-	-	Effects of masitinib on preventing proliferation and migration of macrophage to investigate the association between macrophages and endplates, along with TSCs, in the SOD1G93A plantaris muscles	Treatment with the masitinib significantly reduced infiltration, prevented TSCs loss, and improved reinnervation
Trais 2017 [27]	SOD1G93A rats modeling ALS	-	180–210 days of age, male	Whether the pharmacologic reduction of mast cells by masitinib could modulate the rate of NMJ denervation	Masitinib reduced mast cell, showing 35% decrease in NMJ denervation and reduced motor deficits as compared with vehicle-treated rats. Masitinib also reduced macrophage infiltration, as well as regressive changes in Schwann cells and capillary networks observed in advanced paralysis
Trias 2018 [28]	SOD1G93A rats modeling ALS	-	180–210 days of age, male	Whether masitinib in SOD1G93A rats ameliorates the paralysis progression by modifying mast cell and neutrophil responses	Masitinib prevented mast cell and neutrophil infiltration, axonal pathology, secondary demyelination, and the loss of type 2B myofibers, compared with vehicle-treated rats
Trias 2020 [29]	SOD1G93A rats modeling ALS	-	180–210 days of age, male	Effect of inhibiting CSF-1R and c-Kit by masitinib (30 mg/kg/day) on reducing TSCs reactivity and immune cell infiltration	Treatment with masitinib resulted in a significant decrease in GFAP and S100b immunoreactivity, as compared to vehicle. Masitinib reduced Iba1 + macrophages and c-Kit + mast cells by 60% and 30%, respectively

*ALS*, amyotrophic lateral sclerosis; *rt-PA*, recombinant tissue plasminogen activator; *AD*, Alzheimer’s disease; *ROS*, reactive oxygen species; *TCSs*, terminal Schwann cells; *NMJ*, neuromuscular junctions

### Evidence from the clinical studies

The included clinical studies consisted of five RCTs, along with one post hoc analysis of an RCT and one case report involving a patient from the same RCT. As demonstrated in Table 4, two RCTs were on AD [10, 21], two on MS [8, 22], and one on ALS [9]. The post-hoc analysis study was based on the RCT on ALS, and the case report was also about an ALS patient [23, 24]. In their RCT, Vermersch et al. investigated two doses of masitinib (4.5 and 6.0 mg/kg/day) in patients with primary progressive MS or nonactive secondary progressive MS [22]. They found that masitinib (4.5 mg/kg/day) showed significant benefit over placebo according to the primary endpoint. Also, in the other RCT on MS, masitinib (6.0 mg/kg/day) appeared to have a positive effect on MS-related impairment compared with patients receiving placebo [8]. A phase 2 RCT by Piette et al. investigated masitinib (3.0 or 6.0 mg/kg/day) in patients with AD and found that cognitive decline in the masitinib group was significantly lower than the placebo group [21]. Also, the placebo treatment arm showed a worsening mean regarding the AD functional scales. Dubois et al. confirmed these results in their phase three RCT, as they found significant benefits of masitinib over placebo in terms of all investigated outcomes [10].

**Table 4:** Summary of participants' characteristics, outcomes, and main findings of the included RCTs

Study ID	Country	Sample size (masitinib/placebo)	Participants	Age, mean	Male, *n* (%)	Masitinib dose, mg/kg/d	Outcomes	Efficacy findings	Safety findings
Vermersch 2022 [22]	20 different countries	611 (403/208)	Adult patients (aged 18 to 75) with MS diagnosed according to McDonald’s criteria	49.23	259 (42.4)	4.5 and 6.0	Efficacy outcomes included change from baseline on the EDSS, MSFC, and MSQOL	Masitinib (4.5 mg/kg/d) showed significant benefits over placebo with a δEDSS of 0.001 vs. 0.098, respectively. However, the majority of secondary endpoints did not differ between arms, including the MSFC score (*P* = 0.729), SQOL–physical health (*P* = 0.823), and MSQOL–mental health (*P* = 0.578)	Safety was consistent with masitinib’s known profile (diarrhea, nausea, rash, and hematologic events), with no elevated risk of infection. The rate of serious nonfatal adverse effects was 21.1% for masitinib vs. 12.9% for placebo
Vermersch 2012 [8]	France	35 (27/8)	Adult patients (aged 18 to 60) with RRMS or SPMS	48	17 (49.0)	3.0 and 6.0	Efficacy outcomes included change from baseline on the MSFC and its subcategories	Masitinib showed a positive impact on MS-related impairment for PPMS and SPMS patients. Compared to the placebo group, patients receiving masitinib experienced an improvement in MSFC scores at month-12 (+ 103% ± 189), while the placebo group had a decline in scores (− 60% ± 190)	Safety was consistent with masitinib’s known profile (diarrhea, nausea, rash, and hematologic events) However, a higher incidence of severe and serious events was associated with masitinib
Piette 2011 [21]	France	34 (26/8)	Patients (aged ≥ 50) diagnosed with mild-to-moderate AD	75	13 (38.2)	3.0 and 6.0	The efficacy endpoints were ADAS-Cog, ADCS-ADL, CIBIC-Plus, MMSE, and CDR	Decline of cognitive function, as assessed by ADAS-Cog and MMSE was significantly higher in the placebo group after 12 and 24 weeks; however, no differences were observed regarding the other outcomes	Overall, AEs were more common in the masitinib group compared with the placebo group (65% versus 38%, respectively)
Dubois 2023 [10]	20 different countries	718 (371/277)	Patients with dementia that was probably due to AD (biomarker tests were not required for patient inclusion)	71.7	331 (46.1)	4.5	The primary analysis was the change from baseline on ADAS-cog and ADCS-ADL over 24 weeks	Masitinib showed significant benefit over placebo according to ADAS-cog, with a significant between-group difference of − 2.15 (97.5% CI [− 3.48, − 0.81]); *P* < 0.001. For ADCS-ADL, the between-group difference was 1.82 (97.5% CI [− 0.15, 3.79]); *P* = 0.038	Safety was consistent with masitinib’s known profile (papular rash, neutropenia, hypoalbuminemia)
Mora 2019 [9]	9 different countries	391 (259/132)	Patients (aged 18 to 75) with a laboratory-supported probable, probable, or definite diagnosis of ALS (revised El Escorial criteria)	55.5	244 (62.4)	4.5 and 3.0	The efficacy endpoint was decline in ALSFRS-R, ALSAQ-40, and FVC from baseline to week-48	Masitinib showed a significant benefit over placebo with a decline change difference in ALSFRS-R between the two groups of 3.4 (95% CI 0.65–6.13; *P* = 0.016), corresponding to a 27% slowing in rate of functional decline. ALSAQ-40, FVC, and time-to-event analysis were also significant	Rates of adverse events were 88% with masitinib 4.5 mg/kg/d, 85% with 3.0 mg/kg/d, and 79% with placebo Likewise, rates of serious AE were 31, 23, and 18%, respectively

*MS*, = multiple sclerosis; *EDSS*, Expanded Disability Status Scale; *MSFC*, multiple sclerosis functional composite; *MSQOL*, multiple sclerosis quality of life; *RRMS*, relapsing–remitting multiple sclerosis; *SPMS*, secondary progressive multiple sclerosis; *AD*, Alzheimer’s disease; *ADAS-Cog*, Alzheimer’s Disease Assessment Scale-Cognitive Subscale; *ADCS-ADL*, Alzheimer’s Disease Cooperative Study Activities of Daily Living Inventory; *CIBIC-Plus*, the Clinician’s Interview-Based Impression of Change-Plus caregiver input; *MMSE*, mini-mental state examination; *CDR*, Clinical Dementia Rating; *ALS*, amytrophic lateral sclerosis; *ALSFRS-R*, Revised Amyotrophic Lateral Sclerosis Functional Rating Scale; *ALSAQ-40*, Amytrophic Lateral Sclerosis Assessment Questionnaire 40-item, *FVC*, forced vital capacity

Regarding ALS, a study conducted by Mora et al. examined the effects of masitinib (4.5 or 3.0 mg/kg/day) in ALS patients [9]. The results showed that masitinib significantly slowed down the decline in ALS functional status compared to the placebo group, with a 27% reduction in the rate of functional decline. However, it was noted that the masitinib groups experienced a higher incidence of severe adverse effects. Building upon this RCT, a subsequent long-term survival analysis was carried out to investigate the actual effects of masitinib [23]. The analysis revealed a significant survival benefit of 25 months and a 47% reduced risk of death for patients receiving a dosage of 4.5 mg/kg/day masitinib compared to the placebo group. Additionally, Salvado et al. reported a case of autoimmune-like hepatitis potentially associated with masitinib treatment in one patient from this RCT [24].

## Discussion

### Key findings

The objective of this scoping review was to provide an overview of the existing evidence on the neuroprotective effects of masitinib. Our investigation yielded a total of 14 studies that examined the neuroprotective effects of masitinib, consisting of five RCTs and nine animal studies. Furthermore, we included one post hoc analysis study and one case report, both derived from an included RCT. The collective findings from these studies consistently demonstrated the neuroprotective properties of masitinib. However, the majority of RCTs reported a concerning safety profile for masitinib.

### Interpretation

**Fig. 2:**
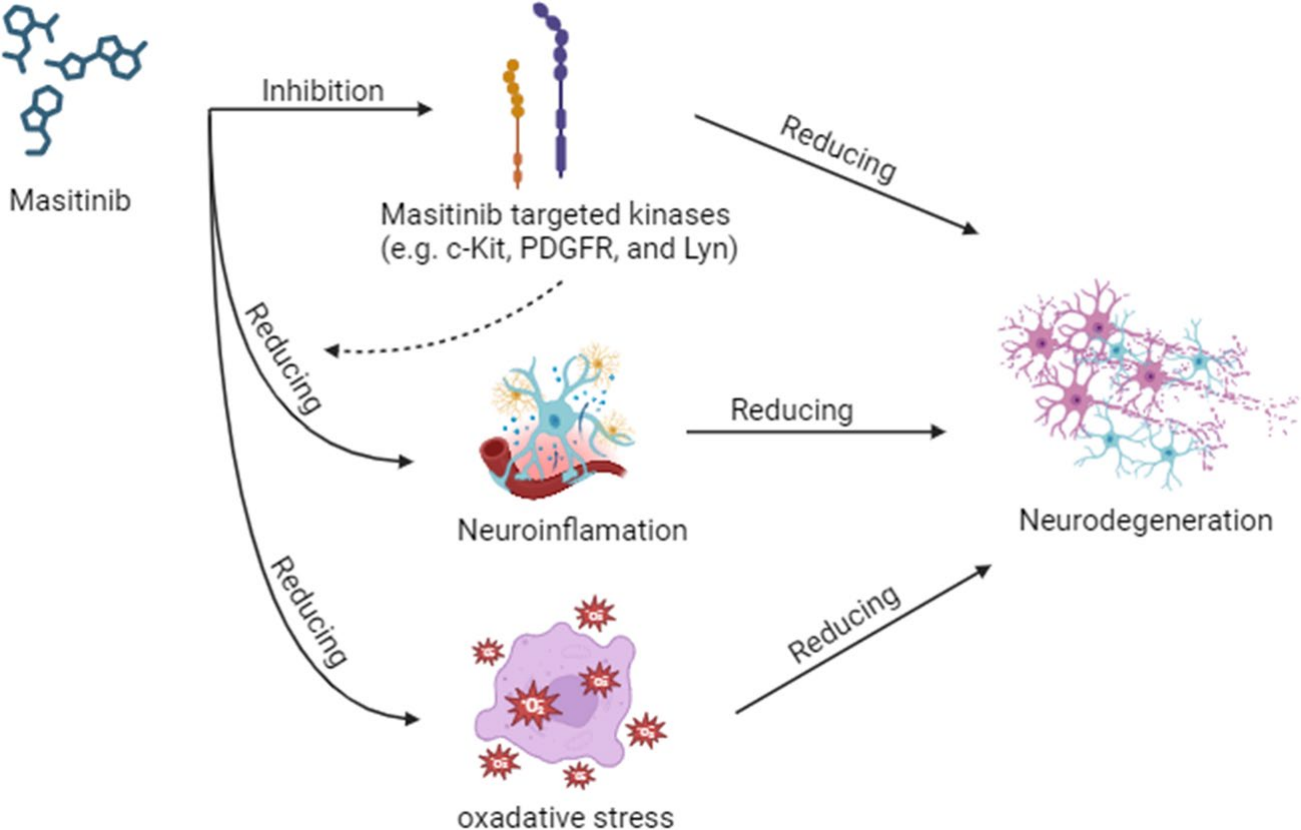
Summarization of the potential neuroprotective mechanisms of masitinib (created with BioRender.com)

Neuroprotection refers to the preservation of neuronal structure and function, aiming to prevent or slow down the progression of neurodegenerative disorders [31]. Various mechanisms have been proposed for neuroprotection, including anti-inflammatory effects, antioxidant properties, modulation of cell survival pathways, and attenuation of excitotoxicity [7, 32]. Masitinib, a tyrosine kinase inhibitor originally developed as an anticancer agent [33], selectively targets specific kinases involved in cell signaling pathways associated with inflammation, cell survival, and tissue remodeling [34]. Masitinib’s first anticancer therapy was approved in canine mast cell tumors and showed promising results [35, 36]. Similarly in humans, masitinib showed significant survival benefit in advanced gastrointestinal stromal tumor and pancreatic cancer [37, 38]. Beyond its oncology applications, masitinib has shown potential benefits in various neurological disorders due to its ability to modulate key pathways implicated in neurodegeneration [11, 26]. Preclinical studies included in our review have demonstrated the neuroprotective mechanisms of masitinib. Firstly, masitinib inhibits certain kinases, including c-Kit, PDGFR, and Lyn, interfering with signaling pathways involved in cell proliferation, survival, and inflammation [34]. Secondly, it reduces neuroinflammation, as demonstrated by Trias et al., who found a reduction in neuroinflammation, microgliosis, and aberrant glial cells associated with masitinib use [26]. Qian et al. also reported alleviated allodynia and decreased inflammatory cytokines in the masitinib-treated group [25]. Thirdly, masitinib exhibits antioxidant activity, as Qian et al. observed a reduction in reactive oxygen species, leading to improved mechanoreception and alleviation of sensory nerve damage [25]. Figure 2 summarizes the potential neuroprotective mechanisms of masitinib.

Although the mechanisms of neurodegeneration are not fully understood, research has confirmed that immunity and inflammation are involved in the pathophysiology of several neurodegenerative diseases, such as ALS, MS, and AD [39–42]. Various immunological cell types, such as cytokines, are expressed and actively present in the brain during neurodegeneration [43]. Cytokines share in repair processes in the central nervous system by facilitating the pathogen clearance and reducing tissue damage [44]. Other cells, such as microglia and astrocytes, are also involved in the immune defense, regulating tissue homeostasis and preserving the brain structure and function [45]. However, it is still not clear whether these immunological cells are beneficial or detrimental in pathological neurological conditions. For example, over expression of cytokines may lead to apoptosis and severe inflammation [46]. Chronic activation of microglia can cause neuronal damage by releasing cytotoxic molecules [47]. Mast cells also exert significant effects on their microenvironment and neighboring cells, including astrocytes, microglia, and neurons, which are implicated in neuroinflammation and neurodegeneration [48]. The involvement of mast cells, cytokines, microglia, and astrocytes in the pathogenesis of neurodegeneration may provide insights into the potential effects of masitinib as a neuroprotective agent. Research has confirmed that masitinib can modify neuroinflammation by reducing mast cells, microgliosis, aberrant glial cells, and inflammatory cytokines [11, 26, 27, 29]. Also, the antioxidant activity of masitinib could be involved in this neuroinflammatory regulation, as oxidative stress has been linked to the progress of several neurodegenerative disorders [49].

In clinical studies included in our review, masitinib has shown promising results in terms of cognitive decline in AD, functional status in ALS, and impairment related to MS [8–10]. In ALS, masitinib targets microglial cells, mast cells, and macrophage infiltration, thereby attenuating neuroinflammatory processes [27, 28]. Also, reducing the reactivity of Schwann cells was addressed as a potential mechanism of masitinib in ALS, contributing to the preservation of neuronal function and slowing down the disease progression [29]. In MS and AD, modulating mast cells’ activity could improve the disruption of the blood–brain barrier and decreases the infiltration of immune cells into the central nervous system, thereby reducing inflammation and preserving neurological function [50]. Overall, masitinib’s mechanism of action in ALS, MS, and AD mostly involves targeting key components of neuroinflammation. Future research is required to enhance our understanding of masitinib’s specific mechanisms in neurodegenerative diseases. Also, future and ongoing RCTs, such as NCT03127267 and NCT05441488, will provide valuable insights and confirm the therapeutic benefits of masitinib.

### Strengths and limitations

This review represents the first comprehensive evaluation of the neuroprotective effects of masitinib. We employed a thorough search strategy and followed established guidelines, ensuring a comprehensive assessment of the available evidence. The review included both preclinical studies and clinical trials, providing a broader perspective on the neuroprotective effects of masitinib. The inclusion of different study types strengthens the overall evidence base. The included studies underwent a methodological quality assessment using appropriate tools, such as RoB 2 for RCTs and the CAMARADES checklist for animal studies. However, our scoping review identified a relatively small number of studies that met the inclusion criteria. This limited pool of evidence may restrict the generalizability of the findings and highlights the need for additional research on the topic.

### Clinical implications and recommendations

Masitinib is a promising and practical treatment for a wide range of neurodegenerative disorders. Multiple pharmacological targets, such as modulating the A and Tau protein signaling cascade and preventing synaptic damage, make it a potential treatment for Alzheimer’s disease-related dementia [51]. Clinical studies have shown that masitinib can slow cognitive decline in patients with mild to moderate Alzheimer’s disease [10]. Furthermore, it may be used as a treatment for progressive forms of multiple sclerosis, since it acts on growth and activation pathways to hinder mast cell survival, migration, cytokine generation, and degranulation [22]. As it targets macrophages, mast cells, and microglia cells, masitinib may be useful in treating ALS because it highlights the disease’s neuroinflammatory activity [9]. Masitinib inhibits the production of inflammatory cytokines, lessens inflammation indirectly, and triggers neuroprotection [52]. While masitinib shows promise in the treatment of various neurodegenerative disorders, further investigation is necessary to address potential adverse effects and optimize its therapeutic use. Continued research and clinical trials will help refine its application and ensure its safe and effective utilization in clinical practice.

## Conclusion

Masitinib shows promising potential as a neuroprotective agent in various neurodegenerative diseases. The available evidence, including preclinical and clinical studies, suggests that masitinib exerts neuroprotective effects through its modulation of key signaling pathways implicated in cell proliferation, survival, neuroinflammation, and antioxidant activity. However, concerns regarding the safety profile of masitinib have been raised. Further research is needed to confirm and explore the therapeutic benefits of masitinib in neurodegenerative diseases. Future studies should focus on addressing the safety concerns associated with masitinib use. Additionally, investigations into optimal dose and potential combination therapies may help maximize the efficacy of masitinib as a neuroprotective agent.

## Data Availability

Not applicable.
